# Regulation of L-Lactate in Glutamate Excitotoxicity Under Cerebral Ischemia: Pathophysiology and Preventive Strategy

**DOI:** 10.3390/ph18070935

**Published:** 2025-06-20

**Authors:** Mao Zhang, Yanyan Wang, Zili Gong, Wen Jiang, Guodong Ge, Hong Guo

**Affiliations:** 1 Department of Medical Genetics, College of Basic Medical Science, Army Medical University, 30 Gaotanyan Main Street, Shapingba District, Chongqing 400038, China; zhangmao2050@163.com (M.Z.); dreamyanyan@126.com (Y.W.); jiangwen0902@163.com (W.J.); gdge@tmmu.edu.cn (G.G.); 2 Department of Neurology, Xinqiao Hospital, Army Medical University, 83 Xinqiao Main Street, Shapingba District, Chongqing 400038, China; gzl_944@163.com

**Keywords:** cerebral ischemia, L-lactate, glutamate, calcium overload, astroglial L-lactate-sensitive receptor (LLR)

## Abstract

Glutamate is an excitatory neurotransmitter in the central nervous system (CNS) that mediates synaptic transmission. However, glutamate homeostasis among neural cells is broken in cerebral ischemia. Excessive glutamate triggers *N*-methyl-d-aspartate receptors (NMDARs) in postsynaptic neurons, leading to intracellular calcium (Ca^2+^) overload and excitoneurotoxicity. At this moment, L-lactate may affect NMDARs and play a protective role in cerebral ischemia. This work proposes that L-lactate regulates glutamate signaling among neural cells. But, dysregulation of L-lactate in glutamate signaling cascades contributes to glutamate excitotoxicity in cerebral ischemia. In detail, L-lactate regulates the glutamine(Gln)-glutamate cycle between astrocytes and presynaptic neurons, which triggers the astroglial L-lactate-sensitive receptor (LLR)-cyclic adenosine monophosphate (cAMP)/protein kinase A (PKA) pathway, coordinating astroglial glutamate uptake and neuronal glutamate transmission. L-lactate mediates glutamate signaling and synaptic transmission among neural cells. In addition, L-lactate promotes the function of mitochondrial calcium uniporter complex (MCUC), which quickly depletes intracellular Ca^2+^ in postsynaptic neurons. In addition, L-lactate can promote the conversion of microglia from the pro-inflammatory (M1) to anti-inflammatory (M2) phenotype. Therefore, regulation of L-lactate in glutamate signaling in the CNS might become a preventive target for cerebral ischemia.

## 1. Introduction

In cerebral ischemia, the rapid release of glutamate and insufficient glutamate uptake result in accumulation of extracellular glutamate, which over-activates glutamate receptors [1]. Metabotropic glutamate receptors (mGluRs) and ionotropic glutamate receptors (iGluRs) are glutamate receptors, whose activity is changed following cerebral ischemia [2]. *N*-Methyl-d-aspartate receptors (NMDARs) are one of the iGluRs, which are ligand-gated and calcium (Ca^2+^)-permeable ion channels, localizing to the pre- and postsynaptic membranes [3]. Overactivation of NMDARs evokes neuronal excitotoxicity. Also, prolonged activation of extrasynaptic NMDARs causes Ca^2+^ overload and neuronal apoptosis [4]. This work primarily discusses glutamate excitotoxicity resulting from NMDAR hyperactivity, and then explores the possibility of regulating glutamate signaling among neural cells in cerebral ischemia.

A clinical trial revealed that glutamate and L-lactate are biomarkers that predict patient death within 3 days after severe head trauma [5,6]. Glutamate excitotoxicity has been extensively discussed, but L-lactate signaling between neural cells and its role in cerebral ischemia have not been discussed. Glutamate is the main excitatory transmitter in the brain. L-lactate not only serves as a source of pyruvate but also as a precursor of glutamate. Importantly, L-lactate significantly reduces the release of glutamate from astrocytes to neuronal synapses [7]. It has been proved that L-lactate is released from astrocytes and oligodendrocytes (OLs) and is used as an energetic substrate of neurons [8]. Even astrocytic L-lactate reduction contributes to neurodegeneration under stroke, suggesting that L-lactate from astrocytes plays a preventive role in stroke [9]. L-lactate is an energetic substrate of neurons and also functions as a signal in the modulation of neuronal excitability, synaptic plasticity, and memory formation through monocarboxylate transporters (MCTs) and G protein-coupled receptors (GPCRs) [10,11]. Specifically, L-lactate produced by glial cells is transported to neurons where lactate stimulates gene expression related to long-term memory formation [11]. Until recently, histone lactylation was observed to activate transcription, which increases glycolytic activity [12]. Hence, L-lactate may play a role as a regulator in glutamate excitotoxicity under cerebral ischemia. This work extensively discusses the regulation of L-lactate in glutamate signaling among neural cells. Importantly, it hypothesizes a mechanism by which L-lactate produced by glial cells (astrocytes, microglia, and oligodendrocytes) alleviates intracellular Ca^2+^ overload induced by NMDAR hyperactivity. Firstly, L-lactate regulates the Glutamine (Gln)-glutamate cycle between astrocytes and neurons. Secondly, L-lactate directly regulates glutamate signaling from presynaptic to postsynaptic neurons. Thirdly, L-lactate promotes mitochondrial calcium uniporter complex (MCUC) in the function of intracellular Ca^2+^ transported into mitochondria in the postsynaptic region. Finally, L-lactate regulates the phenotype of microglia in the response to inflammation.

## 2. Regulation of L-Lactate in Glutamate Excitotoxicity Under Cerebral Ischemia

### 2.1. L-Lactate Regulates Glutamate Metabolism Among Neural Cells

In the brain, glutamate is an important excitatory neurotransmitter that plays an important role in synaptic plasticity [13]. In human brains, there is a high level of glutamatergic activity of synapses and energy demand [14]. Glutamate is released from presynaptic membrane via exocytosis and then triggers glutamate receptors (GluRs) at postsynaptic membrane [15]. Synaptic transmission can be mediated by the activation of NMDARs, which are one of the iGluRs. They are ligand-gated and Ca^2+^-permeable ion channels [16,17]. However, the disturbance of release and reuptake, such as in cerebral ischemia, leads to excessive glutamate accumulation in the synaptic cleft [18]. As a result, NMDARs are over-activated, which induces excitatory neurotoxicity, promoting neuronal death [19]. Evidence suggests that neurons collaborate with astrocytes in the regulation of energy metabolism and neurotransmission [20].

In neurons, which do not contain pyruvate carboxylase to synthesize glutamate, astrocytes must transfer a recyclable substrate via the Gln-glutamate cycle. Glutamate is transported by high-affinity transporters on neurons when it is received by synapses. Gln is absorbed by neurons via high-affinity transporters when it reaches synapses. Upon entering the brain, it is converted to glutamate by phosphate-activated glutaminase [21]. Normally, astrocytes prevent the accumulation of glutamate in extracellular spaces through the absorption of glutamate [22]. In astrocytes, the released glutamate is taken up by glutamate transporter 1 (GLT1) and glutamate-aspartate transporter (GLAST), then converted to Gln through glutamine synthetase (GS). This process not only reduces the excitatory toxicity of neurons, but also promotes glutamate recycling between neurons and astrocytes [23,24]. In addition, glutamate triggers astrocytic mGluRs and the downstream phospholipase C (PLC) cascade, leading to intracellular Ca^2+^ increases. The increased Ca^2+^ promotes the release of gamma-aminobutyric acid (GABA), D-serine, adenosine triphosphate (ATP), and glutamate. Then, these gliotransmitters regulate glutamate release and transmission in presynaptic and postsynaptic neurons [25].

NMDARs have a positive effect on the migration and differentiation of oligodendrocyte precursor cells (OPCs). It is recognized that AMPARs can mediate neuron-oligodendrocyte precursor cell (OPC) synapses [26]. AMPAR-mediated signaling at axon-OPC synapses in the mouse corpus callosum is important for balancing the response of OPCs to proliferation and differentiation cues [27]. Moreover, glutamate is released from neurons, and then stimulates oligodendrocytic NMDARs, which mobilize glucose transporter 1 (GLUT1) into the myelin membrane, which subsequently promotes glucose uptake from the extracellular space [28]. Further, the glucose is converted to L-lactate in OLs, and then the L-lactate is transported into neuronal axons, where it is fasted metabolized to sustain axonal transmission. In addition, L-lactate transport from myelin to axons is mediated by MCTs [29].

Physiologically, microglia are in a “resting” state (M0), which is mediated by “find-me” signals released from neurons [30]. Additionally, glutamate released from the presynaptic compartment triggers microglial GluRs, leading to the release of inflammatory factors [31]. Also, inflammatory factors activate the release of microglial glutamate, stimulating synaptic receptors and altering neurotransmission processes [32] Under pathological conditions, microglia are driven from M0 to the “classically activated” pro-inflammatory (M1) phenotype and the “alternatively activated” anti-inflammatory (M2) phenotype [33]. Microglia in the M2 phenotype promote axonal regeneration, neurogenesis, remyelination, and angiogenesis. However, microglia in the M1 phenotype exacerbate neuronal damage and impede neurogenesis [34]. Hence, a shift of microglia from the M1 to M2 phenotype is beneficial for recovery after stroke [35].

It has been proved that astrocyte-derived L-lactate regulates the glutamate signaling pathway, which is protective against ischemic stroke-induced neurodegeneration [9]. In astrocytes, glutamate activates aerobic glycolysis, which enhances glucose uptake and L-lactate production [36]. Also, L-lactate can regulate glutamate transport between astrocytes and neurons [37]. At post-synapse, glutamate activates NMDARs, which trigger ryanodine receptors (RyRs), leading to Ca^2+^ release from endoplasmic reticulum (ER) [38]. The depletion of the ER Ca^2+^ content is mainly mediated by the mitochondrial matrix through the mitochondrial calcium uniporter complex (MCUC) [39]. MCUC is an evolutionarily conserved calcium channel. MCUC consists of the inner membrane-spanning subunit mitochondrial calcium uniporter protein (MCU), MCU regulatory subunit b (MCUb) and essential MCU regulator (EMRE), and the intermembrane subunits mitochondrial calcium uptake protein 1 (MICU1) and 2 (MICU2). Its function is relevant to bioenergetics, cell death signaling, and the immune system [36]. However, there is no evidence to support the regulation of lactate in MCUC. But, it is recognized that L-lactate upregulates expressions of some reparative genes through lactylation [40,41]. Here, it is hypothesized that L-lactate upregulates MCUC expression through lactylation, which promotes Ca^2+^ uptake in mitochondria.

Although L-lactate has not been used as a drug in the clinical setting, it has been shown that L-lactate has extensive pharmacological effects. Firstly, L-lactate drives cellular deoxyribonucleic acid (DNA) repair capacity, which may promote undesirable alterations in cancer physiology and mitigate retroviral infections [42]. Secondly, L-lactate is a metabolite necessary for multiple functions in the brain and is an alternative energy source during excitotoxic brain injury. Moreover, L-lactate mediates neuroprotection against glutamate-induced excitotoxicity [43]. Importantly, L-lactate modulates astrocytic and microglial inflammation, promotes plasticity-related protein expression, and reduces neurological deficits by potentiating GPR81 signaling in cerebral ischemia [44,45,46]. Hence, L-lactate might become a potential biological drug in brain injury and stroke.

**Figure 1:**
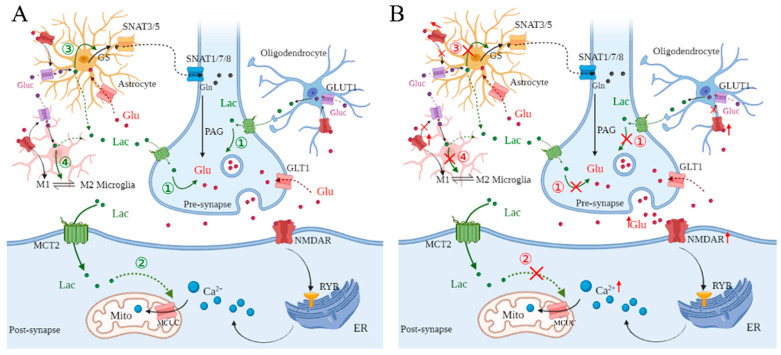
Dysregulation of L-lactate in glutamate metabolism under cerebral ischemia. (**A**) Glutamate (Glu) is an excitatory neurotransmitter, which is released from pre-synapse, and activates *N*-methyl-d-aspartate receptors (NMDARs) at post-synapse. The activation of NMDAR triggers ryanodine receptors (RyRs), leading to calcium (Ca^2+^) release from endoplasmic reticulum (ER). Also, Glu activates NMDARs in astrocytes, microglia, and OLs, promoting the activity of glucose transporter type 1 (GLUT1). As a result, glucose (Gluc) is transported into astrocytes, microglia, and OLs, and then converted to L-lactate. In addition, Glu triggers NMDAR in microglia, leading “resting” state (M0) microglia to convert to the “classically activated” pro-inflammatory (M1) phenotype and the “alternatively activated” anti-inflammatory (M2) phenotype. Normally, the released Glu is taken up by glutamate transporter 1 (GLT1) by pre-synapse and astrocytes. In astrocytes, Glu is converted into glutamine (Gln) through glutamine synthetase (GS), and then transported to neurons by sodium-coupled neutral amino acid transporter (SNAT)3/5 and SNAT1/7/8. In pre-synapse, Gln is converted to Glu by phosphate-activated enzyme glutaminase (PAG). Under physiological conditions, L-lactate mediates glutamate metabolism among neural cells. Firstly, ① L-lactate regulates Glu release from pre-synapse. ② At post-synapse, L-lactate decreases intracellular Ca^2+^ concentration through regulating mitochondrial calcium uniporter complex (MCUC) in inter-membrane of mitochondria (Mito). ③ In astrocytes, L-lactate regulates GS activity, affecting the Glu-Gln cycle. ④ Finally, L-lactate promotes the conversion of microglia from the M1 to M2 phenotype. (**B**) In cerebral ischemia, excessive Glu is released from pre-synapse, which over-activates NMDARs in postsynaptic neurons, leading to intracellular Ca^2+^ overload. Further, overactivation of NMDARs in astrocytes, microglia, and OLs leads to incapability in the regulation of GLUT1, decreasing L-lactate production in astrocytes, microglia, and oligodendrocytes. As a result, ① the release of Glu is dysregulated in presynaptic neurons; ② intracellular Ca^2+^ concentration is overloaded, inducing synaptic injury; ③ the Glu-Gln cycle is inhibited, which may further aggravate glutamate accumulation; ④ conversion of microglia from the M1 to M2 phenotype is suppressed, leading to inflammation.

Conclusively, glutamate is released from pre-synapse, and then activates NMDARs at post-synapse, promising excitatory transmission between neurons. NMDAR activation triggers RYRs of the ER, then leads to the release of Ca^2+^, which is mainly depleted by MCUC in mitochondria. Besides, NMDAR activation promotes glucose transport and L-lactate production in astrocytes, microglia, and OLs. Importantly, the released L-lactate plays a vital role in glutamate signaling among neural cells. At pre-synapse, L-lactate regulates glutamate release. At post-synapse, L-lactate contributes to Ca^2+^ uptake of mitochondria through the regulation of MCUC. In addition, L-lactate regulates the Gln-glutamate cycle in astrocytes and promotes the transformation of microglia from the M1 to M2 phenotype (Figure 1A).

### 2.2. Dysregulation of L-Lactate in Glutamate Metabolism Among Neural Cells Under Cerebral Ischemia

Cerebral ischemia often leads to brain dysfunction and mortality [47]. In cerebral ischemia, glutamate is rapidly released from presynaptic membranes in combination with a deficiency in glutamate uptake, causing accumulation of extracellular glutamate [1], which leads to overactivation of glutamate receptors, including iGluRs and mGluRs [48]. Glutamate accumulation occurs because of increased neuronal release and interrupted astrocyte uptake [49]. As a result, increased excitotoxicity may result from the increase in GS activity. Furthermore, other studies have shown that GS-mediated glutamine synthesis normalizes extracellular glutamate in astrocytes and protects neurons from damage caused by cerebral ischemia [50]. Excess glutamate accumulates quickly at synapses, stimulating NMDARs, which eventually cause neurotoxicity. Thus, the inability of astrocytes in removing excessive glutamate from cellular spaces indirectly aggravates glutamate neuroexcitotoxicity [51]. Moreover, in cerebral ischemia, OPCs migrate and differentiate to OLs in the demyelinated axons, and this process is mediated by glutamate and its receptor NMDARs [52]. Especially, glutamate released from cultured cortical neurons promotes OPC migration via NMDARs by coupling to and activating the T-cell lymphoma invasion and metastasis 1 (Tiam1)/Rac1 pathway [53].

Following stroke, microglia are driven from M0 to the M1 or M2 phenotype [41]. Proinflammatory cytokines secreted by the M1 phenotype of microglia promote glutamate excitotoxicity via hyperexcitation of NMDARs and inhibit synaptic plasticity, leading to neuronal death [54]. Inositol 1,4,5-trisphosphate receptors (IP3Rs) on ER are activated by redox imbalance under stroke, which mobilizes Ca^2+^ into the cytosol. The increased Ca^2+^ signaling in microglia triggers Src at the plasma membrane, which leads to the increased permeability of microglial gap junctions. As a result, glutamate release is promoted during hypoxia [30]. Glutamate can act on both mGluRs and iGluRs of microglia and activate different signaling pathways. Activation of microglial mGluRs suppresses the production of nitric oxide (NO) and reactive oxygen species (ROS) [55,56]. In microglia, mGluRs promote the release of proinflammatory cytokines and trigger the inflammatory process [57,58].

Therefore, in cerebral ischemia, excessive glutamate is released from presynaptic neurons, and then activates NMDARs of postsynaptic neurons, overly promoting synaptic transmission. Additionally, the decreased uptake of glutamate in astrocytes aggravates intercellular glutamate accumulation. Also, NMDAR and AMPAR activation regulates the morphological development of OPCs, but overly triggered NMDARs and AMPARs fail to regulate the morphology, development, and myelination of OPCs [59]. Following excessive glutamate release, microglia are driven from the M0 to M1 phenotype, secreting inflammatory cytokines. Then, these cytokines lead to inflammatory injury of neurons and continuous glutamate release from microglia. Unfortunately, the regulation of L-lactate in glutamate homeostasis among neural cells is impeded, presenting the exacerbation of neuronal damage in cerebral ischemia (Figure 1B).

## 3. Dysregulation of L-Lactate in Postsynaptic Ca^2+^ Concentration Under Cerebral Ischemia

**Figure 2:**
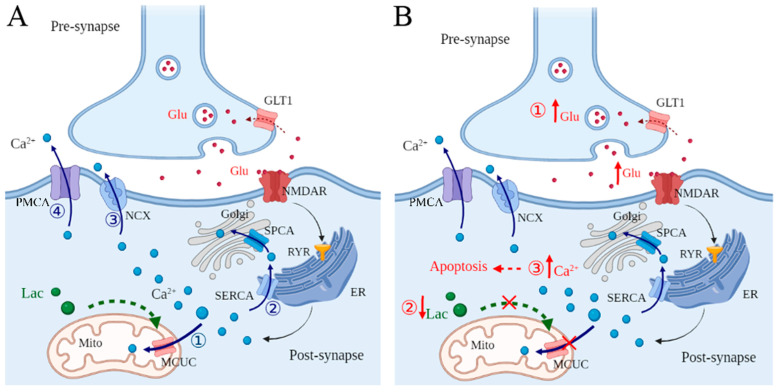
Dysregulation of L-lactate in postsynaptic Ca^2+^ concentration in cerebral ischemia. (**A**) Glutamate (Glu) activates *N*-methyl-d-aspartate receptors (NMDARs) in postsynaptic neurons, which further triggers ryanodine receptors (RyRs) in the endoplasmic reticulum (ER). Then, calcium (Ca^2+^) is released from internal stores in the ER. Normally, the released Ca^2+^ can be regulated by channels and pumps, and the intracellular Ca^2+^ concentration stays at a stable level. ① Most Ca^2+^ is pumped into mitochondria (Mito) through the mitochondrial calcium uniporter complex (MCUC). ② Also, Ca^2+^ is recycled by the ER through sarcoendoplasmic reticulum Ca^2+^-ATPase (SERCA), and then transported into the Golgi apparatus by secretory protein calcium ATPase (SPCA). ③ Na^+^/Ca^2+^ exchanger (NCX) permits Ca^2+^ extrusion from the plasma membrane. ④ Finally, Ca^2+^ is pumped across the plasma membrane by plasma membrane Ca^2+^ transport ATPase (PMCA). Importantly, depletion of intracellular Ca^2+^ is mainly mediated by the mitochondrial matrix through MCUC. (**B**) In cerebral ischemia, ① Glu is excessively released from presynaptic neurons and over-activates NMDARs in postsynaptic neurons. As a result, RyRs are triggered, and excessive Ca^2+^ is released from the ER, increasing the intracellular Ca^2+^ concentration. Particularly, ② the L-lactate released from glial cells is reduced, which may suppress the function of MCUC in the depletion of intracellular Ca^2+^. Thus, ③ intracellular Ca^2+^ is overloaded, leading to apoptosis.

NMDARs are directly coupled to Ca^2+^ channels, the activation of which determines cytosolic Ca^2+^ influx. IP3Rs regulate Ca^2+^ release from the ER to the intracellular compartment [60,61]. Further, IP3Rs can be activated by NMDARs through the triggering of large-conductance cation channels [62]. In addition, RyRs play a role similar to that of IP3Rs in the regulation of Ca^2+^ release from the ER [63]. Normally, the intracellular Ca^2+^ concentration can be regulated by channels, pumps, and buffering systems. Sarcoendoplasmic reticulum Ca^2+^-ATPase (SERCA) pumps Ca^2+^ into the ER, and then Ca^2+^ is transported into the Golgi apparatus via secretory protein calcium ATPase (SPCA) [64]. Furthermore, Ca^2+^ can be pumped across the plasma membrane with the assistance of plasma membrane Ca^2+^ transport ATPase (PMCA) [65], and Ca^2+^ is transported without energy consumption through the Na^+^/Ca^2+^ exchanger (NCX) [66]. MCUC mediates Ca^2+^ entry into the mitochondria, which regulate the signaling pattern of intracellular Ca^2+^ [36]. Significantly, L-lactate-derived lactylation of histone lysine residues serves as an epigenetic modification that directly stimulates gene transcription from chromatin [67]. Here, it is suggested that L-lactate may upregulate MCUC, which promotes Ca^2+^ entry into the mitochondria, lowering the intracellular Ca^2+^ concentration (Figure 2A).

The ER lumen is the major Ca^2+^ storage compartment, and depletion of the ER Ca^2+^ content is followed by rapid accumulation inside the mitochondrial matrix through MCUC. The close proximity between the ER and mitochondria comprises mitochondrial reticular and ER networks, which are termed mitochondria-associated membranes (MAMs) [68]. In cerebral ischemia, NMDAR overactivation induces ER stress, and the quantity of ER-mitochondria connections is significantly increased, which promises Ca^2+^ uptake in mitochondria and ATP production [69]. In ER stress, IP3Rs or RyRs are triggered by NMDAR overactivation, facilitating the release of Ca^2+^ from the ER. In MAMs, voltage-dependent anion channels (VDACs) form large voltage-gated pores in the outer mitochondrial membrane at the ER–mitochondria contacts, which transfer the released Ca^2+^ to the mitochondrial intermembrane space [70]. To reach the mitochondrial matrix, the Ca^2+^ located inside the intermembrane space must pass through the MCUC, which rapidly decreases the intracellular Ca^2+^ concentration [71].

NMDARs are over-activated, which leads to cytosolic Ca^2+^ influx and activation of IP3Rs and RyRs, resulting in an increase in the intracellular Ca^2+^ concentration [72]. This overload of intracellular Ca^2+^ leads to necrosis or apoptosis, resulting in fragmentation of deoxyribonucleic acid (DNA), degraded cross-linking and cytoskeletal proteins, apoptosis, and phagocytosis [60,73]. Additionally, the decreased L-lactate supplement from glial cells leads to MCUC dysfunction, which accentuates cytosolic Ca^2+^ overload in cerebral ischemia (Figure 2B).

## 4. L-Lactate Triggers Astroglial LLR-cAMP/PKA Pathway, Promoting Glycolysis and Glutamate Uptake in Astrocytes

Reduction of astrocytic L-lactate contributes to neurodegeneration under stroke. Meanwhile, L-lactate plays a preventive role in cerebral ischemia [9]. Blood-derived glucose is transported into astrocytes, then decomposed into L-lactate. L-lactate is supplied to neurons, where it serves as an energy source [74]. Further, it is suggested that astrocyte-derived L-lactate is required for numerous neuronal cell functions [10]. L-lactate functions as a signaling molecule by serving as an agonist for GPCRs [75]. Specific GPCRs of lactate include G-protein-coupled receptor 81 (GPR81), neuronal L-lactate-sensitive receptor (LLR), astroglial unidentified LLR, L-lactate-sensitive olfactory receptor 78 (Olfr78), and proton-sensitive G-protein coupled receptor 4 (GPR4) [9,76]. GPR81 couples to Gi/0-proteins, leading to cAMP downregulation; LLR and Olfr78 couple to Gs-proteins and increase cAMP production; and GPR4 takes part in presumable allosteric modulation. However, L-lactate at physiological levels can hardly activate GPR81, which is normally excited during exercise or pathology [77]. Astroglial LLR and its downstream cAMP signaling pathway are triggered at a physiologic L-lactate concentration, which might contribute to neurodevelopmental disorders or ischemic stroke-induced neurodegeneration [9,78].

Glutamate uptake of astrocytes activates astrocytic aerobic glycolysis, leading to glucose uptake and lactate release [36]. Astrocytes generate glycolytically derived L-lactate and L-serine, which are transported into neurons, sustaining neuronal energy needs and modulating neurotransmitter-receptor activity [79]. Disruption of this metabolic coupling may contribute to the progression of neurological diseases, including cerebral ischemia [44]. L-lactate can be covalently modified to lysine residues of histones, a process known as lactylation [67]. This histone lactylation provides a molecular mechanism for lactate in physiological or pathological processes, such as metabolic disability or ischemic injury [80,81]. In cerebral ischemia, enriched lactate induces histone lactylation in high mobility group box 1 (HMGB1) and promotes HMGB1 expression. HMGB1 leads to pyroptosis in cerebral ischemia [81]. Pyroptosis is a pro-inflammatory death mode. It has been suggested that the pyroptosis pathway might be a novel treatment target for cerebral ischemia, but rigorous experimental evidence is lacking [82].

**Figure 3:**
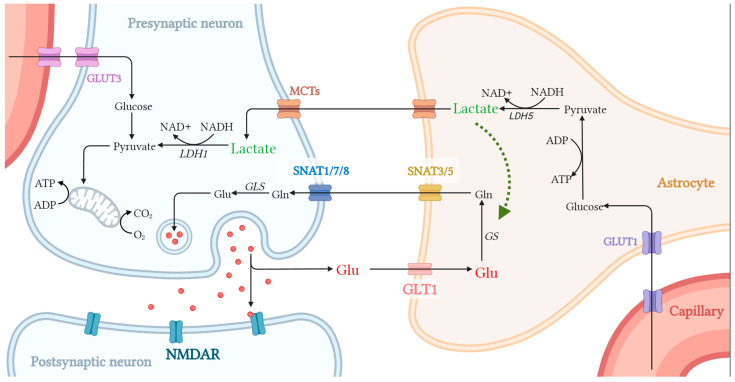
L-lactate regulates the glutamate shuttle between astrocytes and neurons. Glucose is transported into astrocytes and neurons from capillary through glucose transporter 1 (GLUT1) and glucose transporter 1 (GLUT3), respectively. In neurons, glucose is mainly metabolized through aerobic oxidation. In astrocytes, glucose is catalyzed to L-lactate via glycolysis, and then transported into neurons via monocarboxylate transporters (MCTs) and used as an energetic substrate. Further, L-lactate regulates the glutamine (Gln)-glutamate cycle between astrocytes and neurons. In this cycle, astrocytes uptake extracellular glutamate via glutamate transporter 1 (GLT1), which is then converted to Gln via glutamine synthetase (GS). Then, Gln is transferred to presynaptic neurons with the assistance of sodium-coupled neutral amino acid transporter (SNAT)3/5 and SNAT1/7/8. Further, Gln is metabolized to glutamate by glutaminase (GLS), is then released from presynaptic neurons, and binds to *N*-methyl-d-aspartate receptors (NMDARs) at postsynaptic neurons, promising excitatory neurotransmission.

However, malignant astrocyte swelling and impaired glutamate clearance are found in cerebral ischemia [83]. In physiology, the Gln-glutamate cycle controls glutamate metabolism. In this cycle, astrocytes uptake extracellular glutamate through GLT1, and then glutamate is converted to Gln via GS in astrocytes. Gln is transferred to the extracellular milieu via sodium-coupled neutral amino acid transporter (SNAT)3/5, and then into presynaptic neurons via SNAT1/7/8 [84]. In presynaptic neurons, Gln is converted into glutamate by glutaminase (GLS) [85]. Rapid uptake and glutamate conversion in astrocytes prevent prolonged activation of glutamatergic receptors in postsynaptic neurons. Once this process is inhibited or unchecked, it will trigger excitotoxicity in neurons. Hence, the extracellular glutamate must be promptly removed by astrocytes in cerebral ischemia (Figure 3).

**Figure 4:**
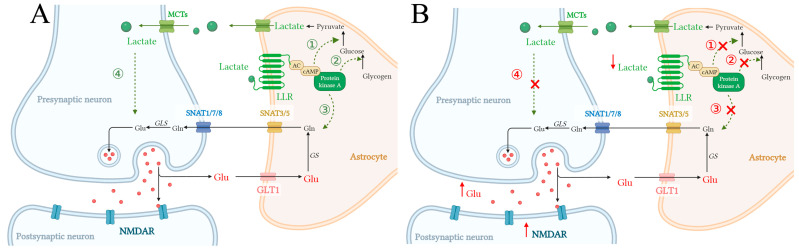
L-lactate regulates astroglial glutamate uptake and glycolysis through the L-lactate-sensitive receptor (LLR)-cyclic adenosine monophosphate (cAMP)/protein kinase A (PKA) pathway. (**A**) Astroglial L-lactate is transported into the intercellular space via monocarboxylate transporters (MCTs). L-lactate triggers LLR, which couples Gs-proteins and activates the adenylate cyclase (AC)/cAMP/PKA pathway. This signaling ① promotes glycogen degradation, ② glycolysis, and increased L-lactate production. Importantly, L-lactate regulates ③ glutamate uptake of astrocytes and ④ neuronal glutamate transmission by mediating the glutamine (Gln)-glutamate (Glu) cycle between astrocytes and neurons. (**B**) In cerebral ischemia, the regulation of L-lactate and its downstream LLR-cAMP/PKA signaling pathway is impeded, which aggravates neuronal glutamate excitotoxicity.

Astroglial LLR couples to Gs-proteins and triggers the cAMP/PKA pathway. Meanwhile, cAMP/PKA signaling facilitates glutamate uptake into cells [86]. In the CNS, L-lactate secreted by astrocytes is released through MCTs and binds to astroglial LLR, stimulating adenylate cyclase (AC) and cAMP production. This activation of the cAMP/PKA pathway promotes glycogen degradation, glycolysis, and increased L-lactate production [75]. In addition, this L-lactate-positive feedback in astrocytes ensures an L-lactate concentration gradient between astrocytes and neighboring cells, maintaining cellular activity, such as the regulation of L-lactate in glutamate excitotoxicity. Here, it is suggested that L-lactate triggers the astroglial LLR-cAMP/PKA signaling pathway, which promotes extracellular astroglial glutamate uptake and alleviates neuronal glutamate excitotoxicity (Figure 4A). However, this L-lactate-glutamate coupling loop might be inhibited in cerebral ischemia. In that scenario, L-lactate can hardly activate the astroglial LLR-cAMP/PKA signaling pathway, which leads to reduced glutamate uptake by astrocytes. As a result, the extracellular lactate concentration is increased and over-activates NMDAR in postsynaptic neurons, leading to neuronal excitotoxicity (Figure 4B).

## 5. Discussion

Glucose is the obligatory fuel for brain cells; it is metabolized mostly by aerobic glycolysis and rarely by anaerobic glycolysis [37]. In the CNS, L-lactate is mainly derived from astrocytes and OLs, and is further utilized by neurons [87]. Astrocyte–neuron L-lactate flow was primarily discussed in a scheme of glutamate-induced glycolysis in astrocytes during physiological activation [88]. In detail, glutamate depolarizes neurons through its receptors, which can be terminated by an efficient glutamate uptake system in astrocytes [89]. Especially, glutamate-cotransported Na^+^ can activate Na^+^/K^+^ ATPase, which fuels glycolytic enzymes and stimulates glycolysis, producing L-lactate in astrocytes. L-lactate, once released from astrocytes, can be recycled by neurons [90]. L-lactate is recognized as an energetic metabolite that is transferred from astrocytes, microglia, and OLs to neurons, supporting neuronal transmission [91]. Until recently, it was not known that L-lactate regulates gene expression through histone lactylation [65]. Here, this work reveals that L-lactate regulates glutamate signaling among neural cells. But, under cerebral ischemia, the role of L-lactate in glutamate signaling is suppressed, which exacerbates glutamate excitotoxicity.

In cerebral ischemia, the concentration of extracellular glutamate is rapidly increased [19]. Accumulated glutamate in the synaptic cleft triggers neuronal death, causing mental or physical handicap [92]. Glutamate accumulation in the synaptic cleft induces excessive NMDAR activation at the postsynaptic region [93]. Ca^2+^ signals induced by synaptic NMDAR regulate neuronal plasticity, and Ca^2+^ is released from ER following the activation of IP3Rs or RYRs [94]. Meanwhile, excessive glutamate-induced NMDAR hyperactivity promotes Ca^2+^ release from the ER, leading to intracellular Ca^2+^ overload [95]. Intracellular Ca^2+^ accumulation leads to excitotoxicity, apoptosis, and cell death [96]. Meanwhile, L-lactate plays a role as a positive modulator of NMDAR-mediated signaling in plasticity gene expression and memory consolidation [97].

This study postulates that L-lactate is protective against glutamate excitotoxicity under physiological conditions. L-lactate functions as a signal molecule, transported from glial cells to neurons. However, this process is suppressed under cerebral ischemia, leading to dysregulation of L-lactate in the glutamate signaling pathway among neural cells. As a result, glutamate transmission in synapses is abnormal, and then NMDARs are hyperactivated. NMDAR overactivation at synapses triggers RYRs, which promote Ca^2+^ release from the ER. Physiologically, MCUC largely depletes most of the released Ca^2+^. But, in cerebral ischemia, MCUC function is inhibited following L-lactate dysregulation, and intracellular Ca^2+^ overload leads to apoptosis. Additionally, L-lactate regulates glutamate transmission from presynaptic neurons to postsynaptic neurons in an astrocyte–neuron lactate shuttle (ANLS) shuttle. However, this L-lactate-glutamate coupling mode is disabled in cerebral ischemia. As a result, glutamate is accumulated in neurons, which over-activates NMDARs and causes an overload of intracellular Ca^2+^, inducing neuronal excitotoxicity. Therefore, targeting L-lactate metabolism and its regulation in glutamate signaling in neural cells could become a promising preventive strategy for cerebral ischemia.

## Data Availability

Not applicable.

## Abbreviations

The following abbreviations are used in this manuscript:

CNS	Central nervous system
NMDARs	*N*-Methyl-D-aspartate receptors
Ca^2+^	Calcium
Glu	Glutamate
Gln	Glutamine
Mito	Mitochondria
MCUC	Mitochondrial calcium uniporter complex
LLR	L-lactate-sensitive receptor
cAMP	Cyclic adenosine monophosphate
PKA	Protein kinase A
iGluRs	Ionotropic glutamate receptors
mGluRs	Metabotropic glutamate receptors
OLs	Oligodendrocytes
MCTs	Monocarboxylate transporters
GPCRs	G protein-coupled receptors
GluRs	Glutamate receptors
AMPARs	A-amino-3-hydroxy-5-methyl-4-isox-azolepropionic acid receptors
GLT1	Glutamate transporter 1
GLAST	Glutamate-aspartate transporter
GS	Glutamine synthetase
PLC	Phospholipase C
ATP	Adenosine triphosphate
GABA	Gamma-aminobutyric acid
OPCs	Oligodendrocyte precursor cells
OPC	Oligodendrocyte precursor cell
GLUT1	Glucose transporter 1
M0	A “resting” state
M1	“Classically activated” pro-inflammatory
M2	“Alternatively activated” anti-inflammatory
RyRs	Ryanodine receptors
ER	Endoplasmic reticulum
Tiam1	T lymphoma invasion and metastasis 1
IP3Rs	Inositol 1,4,5-trisphosphate receptor
NO	Nitric oxide
ROS	Reactive oxygen species
SERCA	Sarcoendoplasmic reticulum Ca^2+^-ATPase
SPCA	Secretory protein calcium ATPase
PMCA	Plasma membrane Ca^2+^ transport ATPase
NCX	Na^+^/Ca^2+^ exchanger
MAMs	Mitochondria-associated membranes
VDACs	Voltage-dependent anion channels
DNA	Deoxyribonucleic acid
Olfr78	L-lactate-sensitive olfactory receptor 78
GPR4	G-protein coupled receptor 4
HMGB1	High mobility group box 1
SNAT	Sodium-coupled neutral amino acid transporter
GLS	Glutaminase
AC	Adenylate cyclase
ANLS	Astrocyte–neuron lactate shuttle
